# Induction heating applied to anisole HDO using formic acid as a hydrogen source

**DOI:** 10.1039/d5cy00707k

**Published:** 2025-10-08

**Authors:** Verónica Naharro-Ovejero, Mónica Dhanjani, Gorka Salas, Ana Belén Dongil

**Affiliations:** a Instituto de Catálisis y Petroleoquímica (CSIC), Campus Universitario de Cantoblanco 28049 Madrid Spain a.dongil@csic.es; b Instituto Madrileño de Estudios Avanzados en Nanociencia, Campus Universitario de Cantoblanco 28049 Madrid Spain; c Unidad de Nanomateriales Avanzados, IMDEA Nanociencia (Unidad de I + D + I Asociada al Instituto de Ciencia de Materiales de Madrid, CSIC) 28019 Madrid Spain; d Unidad Asociada de Nanobiotecnología (CNB-CSIC e IMDEA Nanociencia) 28049 Madrid Spain

## Abstract

Magnetically induced heating catalysis using encapsulated magnetic nanoparticles as heating agents presents itself as a new efficient method for carrying out high-temperature reactions. In this work, magnetic Fe, Co, and FeCo nanoparticles encapsulated in carbon were synthesized using various methods. Rhenium oxide supported on high-surface-area graphite was used as a catalyst for the gas-phase HDO reaction of anisole, a model molecule for HDO studies of biomass-derived compounds. Characterization confirmed the formation of metallic nanoparticles, the alloying of FeCo and the successful coating with a graphitic-like carbon film around the NPs, resulting in core–shell type materials. According to the catalytic results, the activity and the selectivity were similar when using formic acid (FA) or hydrogen (H_2_). Furthermore, by comparing the use of conventional and magnetic heating, it was concluded that carbon encapsulation is an effective strategy to generate a bed that heats but does not catalyze. The ReO_*x*_ catalyst stood out for its capacity to break the OCH_3_ bond, forming benzene as the major product. Among the different MNPs, FeCo@CHT presented the best properties and performance.

## Abstract 2

Magnetically induced heating catalysis using encapsulated magnetic nanoparticles as heating agents presents itself as a new efficient method for carrying out high-temperature reactions.

## Graphical abstract

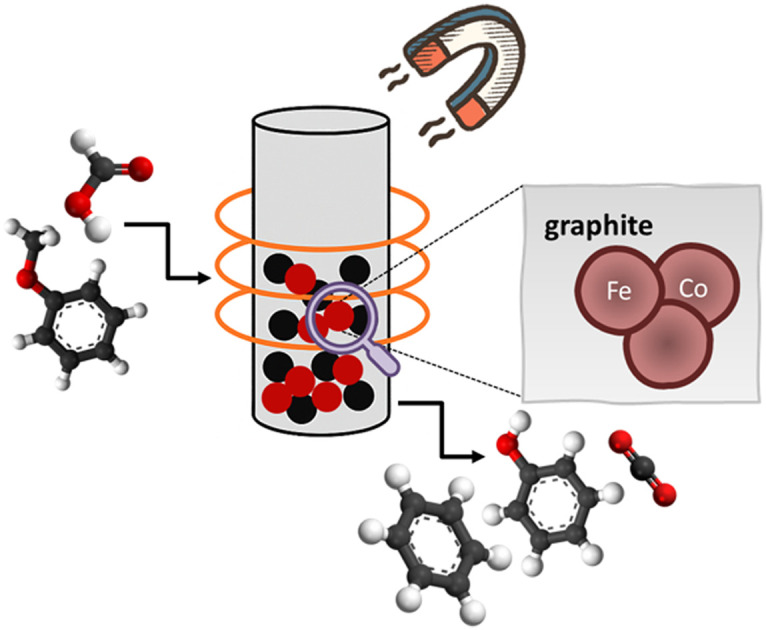

## 1. Introduction

Nowadays, the treatment of biomass for its transformation into chemicals and fuels is emerging as a great alternative to the traditional fossil fuel model, which currently accounts for approximately three-quarters of the global energy supply.^1^ More specifically, the treatment of lignocellulosic biomass presents itself as an environmentally friendly alternative.^2,3^ Among its components (hemicellulose, cellulose, and lignin), lignin is interesting, as it is the only constituent composed of polymeric aromatic chains.^4,5^ These chains can be broken down through processes such as pyrolysis.^6^ However, the resulting products are rich in oxygen (over 40 wt%) and must be processed to obtain high-value compounds, such as bio-oil and fine chemicals.^7^ Hydrodeoxygenation (HDO) is the process used to remove oxygen, and in the laboratory, model molecules such as anisole^8,9^ or guaiacol^10,11^ are used to study the process, with appropriate catalysts and conditions of high temperature and pressure.^12^ The HDO process is highly hydrogen demanding and around 94% of hydrogen is currently obtained from fossil sources.^13^ Utilizing molecules derived from various biomass treatments as a hydrogen source represents a sustainable and innovative alternative for biorefineries. This has led to the use of molecules such as alcohols^14^ or formic acid,^15^ a by-product of cellulose hydrogenolysis^16^ as hydrogen donors in different types of reactions in liquid^17^ and gas phase.^18^ Formic acid (FA) is the simplest carboxylic acid, with a specific energy of 5.3 MJ kg^−1^, corresponding to a relatively high hydrogen content (4.4 wt%).^15^ Additionally, FA exhibits low flammability and low toxicity and is stable at room temperature.^19^ The decomposition of FA can follow two competing pathways: dehydrogenation (Δ*G*° = −32.9 kJ mol^−1^; Δ*H*° = 31.2 kJ mol^−1^), producing H_2_ and CO_2_, or dehydration (Δ*G*° = −12.4 kJ mol^−1^; Δ*H*° = 29.2 kJ mol^−1^), yielding H_2_O and CO. Both reactions can occur simultaneously, so if hydrogen production is the primary goal, dehydration must be minimized, also preventing carbon monoxide formation which can poison the catalyst, leading to lower efficiency of the process. As previously discussed, HDO reactions require elevated temperatures due to their endergonic nature and/or high activation energy barriers.^20^ To address these issues, including the potential for energy savings, a novel approach in the field of heterogeneous catalysis has emerged: magnetic heating. This technique, originally applied in cancer therapy (magnetic hyperthermia),^21,22^ is based on the principle that ferromagnetic materials generate heat through relaxation or hysteresis losses when exposed to a high-frequency alternating magnetic field.^23,24^ The primary advantage of magnetic induction heating is its ability to deliver localized heating directly to the catalytic bed without heating the entire reactor,^25–27^ resulting in faster system response times, improved energy efficiency, and greater control over reaction conditions.^20^ Moreover, induction heating can induce localized heating on the nanoparticles' surface while keeping the medium relatively cold. This is beneficial for the operation of systems in outside conditions, which is useful in systems that consist of endothermic and exothermic steps. In our case, the localized heating is useful for selectively promoting the conventional endothermic pathway. Moreover, magnetic heating also has advantages in exothermic reactions, since it can help start exothermic reactions by providing rapid, localized heat to overcome the activation energy barrier. Unlike conventional heating, it directly warms conductive materials or catalysts, allowing precise control and efficient initiation. Once the reaction becomes self-sustaining, the induction can be switched off, avoiding excess heat and improving safety and energy efficiency.^28^

The heating efficiency of these materials is typically characterized by their specific absorption rate (SAR).^29^ In heterogeneous catalysis, catalysts with a high SAR would offer several advantages: (i) near-instantaneous heating to rapidly initiate or stop reactions, (ii) eliminating the need to heat the entire reactor and focusing solely on the catalytic bed and (iii) energy transfer would occur *via* induction, a process that is more efficient than conduction, as heating would originate within the material itself rather than from the exterior of the reactor.^30^ However, it should be taken into account that magnetic nanoparticles, in addition to generating heat, may also catalyze reactions to undesired products or suffer modifications on the magnetic properties after prolonged use in reactions. To avoid these drawbacks, one way to ensure that nanoparticles act solely as heating agents is by coating them with carbon^31^ or silica.^32^ Carbon coatings, in particular, enhance the sintering resistance of these materials due to their high thermal stability and strong confinement properties.^31^ This novel heating method has been scarcely used for HDO reactions of biomass molecules using iron carbide^33^ or iron- and cobalt based-nanomaterials as heating material.^34,35^ Recently, Mustieles Marin *et al.*,^36^ carried out the HDO of acetophenone and furfural through magnetic induction heating in the liquid phase. They employed nanoparticles with a core composed of a Ni–Fe alloy and a Ni-enriched surface as both the heating material and catalyst. Under reaction conditions of 3 bar H_2_ pressure and a magnetic field of 49 mT, they achieved complete conversion for acetophenone and partial conversion for furfural (60–70%). For this study, hydrogenation of anisole with formic acid was carried out under magnetic induction heating in the gas phase, using rhenium oxide supported on high-surface-area graphite as a catalyst. Rhenium is a relatively rare transition metal known for its excellent catalytic properties,^37^ particularly in hydrogenation and hydrodeoxygenation reactions.^38–42^ When supported on materials with high surface area and conductivity, such as graphitic carbon, rhenium oxide can exhibit enhanced catalytic performance.^43^ Graphitic materials provide several advantages as catalyst supports. Their high thermal and electrical conductivity combined with a large specific surface area enables excellent dispersion of the active metal species, improving the overall catalytic activity.^44^ In addition, graphite exhibits high stability under acidic conditions.^45^ We investigated a system in which the catalyst is mixed with the heating agent in order to combine the heating capacity of the coated metallic nanoparticles based on Fe and Co with the properties of supported rhenium as a catalyst. With this aim, several synthetic procedures for magnetic nanoparticles have been investigated.

## 2. Experimental

### 2.1 Catalyst preparation

Re-based catalysts were synthesized on a commercial high-surface-area graphite (G, Timcal, *S*_BET_: 400 m^2^ g^−1^) using the incipient wetness impregnation method. A 50 : 50 vol% aqueous ethanol solution of NH_4_ReO_4_ was employed to achieve a 10 wt% Re loading, followed by drying at 100 °C for 12 h. The resulting material was activated as described in the catalytic reaction experimental section.

### 2.2 Magnetic nanoparticle preparation

#### Hydrothermal synthesis

Graphite-encapsulated metal core–shell nanoparticles were synthesized through a hydrothermal process followed by heat treatment, a variation of that reported by Lee *et al.*^46^ A solution consisting of a metal source (Fe(NO_3_)_3_·9H_2_O = 5 mmol and Co(NO_3_)_2_·6H_2_O = 5 mmol) and glucose (C_6_H_12_O_6_ = 8 mmol) was stirred in water until the solution became clear. The mixture was then transferred to a 100 mL stainless steel autoclave and heated at 190 °C for 9 h (pressure of 15 bar). The products were washed several times with distilled water, filtered off, and finally dried in an oven at 100 °C for 12 h. Finally, the dried products were heat-treated at 800 °C for 3 h under a He atmosphere to grow a graphite shell on the surface of the metal nanoparticles. The three types of MNPs were labelled Fe@C_HT_, Co@C_HT_ and FeCo@C_HT_.

#### Coprecipitation synthesis

Maghemite nanoparticles were synthesized by a modified Massart coprecipitation method^47^ using FeCl_3_ and FeCl_2_ (2 : 1) as precursors and NH_4_OH (25%) to form an alkaline solution. The resulting Fe_3_O_4_ nanoparticles were converted to maghemite (γ-Fe_2_O_3_) through a thermal acid treatment. After the synthesis, the nanoparticles underwent surface modification with dextran (40 kD) as a carbon source and heat-treated at 800 °C for 3 h with He. The resulting material was named Fe@C_CP_.

#### Impregnation synthesis

Commercial nanoparticles (Sigma-Aldrich CAS-No: 1317-61-9) of Fe_3_O_4_ were encapsulated using a simple impregnation method and posterior thermal treatment. Glucose was dissolved in water and impregnated in a Fe_3_O_4_ surface (molar ratio of 1 : 1) and then dried at 100 °C for 12 h. Finally, the resulting product was heat-treated under the same conditions as in the hydrothermal synthesis to carry out the graphite encapsulation. This material was denoted as Fe@C_I_.

### 2.3 Characterization

X-ray diffraction (XRD) patterns were recorded using a polycrystal X' Pert Pro PANalytical diffractometer with Ni-filtered Cu Kα radiation (*λ* = 1.54 Å). The measurements were conducted at 45 kV and 40 mA, covering a 2*θ* range of 4° to 85° with a step size of 0.04° s^−1^.

X-ray photoelectron spectroscopy (XPS) was performed using non-monochromatic Al Kα radiation (200 W, 1486.61 eV) with a SPECS GmbH UHV system and a PHOIBOS 150 9MCD energy analyzer. Survey spectra were obtained with a 50 eV pass energy, and region spectra with a 20 eV pass energy. Binding energies were referenced to the C 1s peak of graphitic carbon at 284.6 eV, with an equipment error of less than ±0.01 eV.

Further structural characterization was conducted using high-resolution transmission electron microscopy (HRTEM). Both images and electron diffraction (ED) patterns were captured with a JEOL JEM 3000F microscope operating at an acceleration voltage of 300 kV. Samples were prepared by crushing the powders in ethanol and dispersing them onto copper grids coated with a holey carbon film. Additional chemical composition analysis was performed using energy-dispersive X-ray spectroscopy (EDX) within the same JEOL JEM 3000F microscope.

Thermogravimetric analysis (TGA) was carried out in a TA Instruments TGA 500 analyzer with a heating rate of 10 °C min^−1^ in an air atmosphere from room temperature to 850 °C.

Vibrating sample magnetometry (VSM) was performed using an MLVSM9 Mag Lab 2 T instrument (Oxford Instruments). Samples were precipitated, dried, weighed, and placed in gelatin capsules. After saturating the samples to a 2 T magnetic field, magnetization (*M*) *vs.* applied magnetic field (*H*) curves were acquired at room temperature. Saturation magnetization (*M*_S_), expressed in emu per g of material, was determined from the experimental *M*(*H*) data in the high-field region, where magnetization exhibits a linear relationship with 1/*H*. These values were extrapolated to an infinite field (1/*H* = 0). The mass of magnetic material was calculated by correcting the sample weight based on thermogravimetric analysis (TGA) data.

Qualitative and quantitative analyses using TXRF were conducted using the S2 PicoFox TXRF spectrometer, a benchtop model from Bruker Nano based in Germany. This apparatus featured a Mo X-ray source functioning at 50 kV and 600 μA along with a multilayer monochromator that achieved 80% reflectivity at 17.5 keV (Mo Kα). It also incorporated an XFlash SDD detector, boasting an effective area of 30 mm^2^ and an energy resolution superior to 150 eV for 5.9 keV (Mn Kα). The deconvolution and integration of data were managed with the Spectra v. 7.5.3 software package provided by Bruker. For the sample digestions, two different systems were utilized. The first method involved an open vessel digestion using the DigiPREP block digestion system from SCP Science in Canada, which operates at temperatures of up to 180 °C and includes a graphite heating block for processing 24 reactors containing 50 mL Falcon-type tubes. The second method employed high-pressure and temperature microwave acid digestion technology utilizing the UltraWAVE digestion system from Milestone, Italy, which features a single reaction chamber capable of functioning at pressures reaching 199 bar and temperatures of up to 270 °C.

Temperature-programmed desorption (TPD) was employed to study the adsorption of reactants in the spent catalysts. The experiments were performed in an ultrahigh vacuum (UHV) apparatus equipped with a UTI quadrupole mass spectrometer (Prisma Pro QMG 250). The TPD experiments were carried out in a fixed catalytic reactor system by placing 0.1 g of catalyst and a thermocouple in the middle of the catalyst bed and applying a temperature ramp of 5 °C min^−1^ to 400 °C in He (99.999%).

### 2.4 Catalytic reaction

Gas-phase anisole hydrodeoxygenation was performed at atmospheric pressure in a fixed-bed glass reactor (int. d. 1.3 cm). The sample (285 mg) was charged in the reactor and was then treated at 350 °C (5 °C min^−1^) in a He (99.999%, 50 ml min^−1^) or H_2_ atmosphere (99.999%, 50 ml min^−1^) for 1 (under H_2_) or 2 h (under He) to obtain the ReO_*x*_/G and/or Re/G catalyst, respectively.

The reagent mixture (anisole–FA mol ratio 1 : 6) was added using a syringe pump (IPS-14S Independent Double Channel Syringe Pump) with a total flow of 1.38 ml min^−1^, diluted in 50 ml min^−1^ of helium flow (99.999%) and a gas hourly space velocity of 2 h^−1^. All flow lines were heated at 100 °C. The HDO reaction was carried out in two different ways. Initially, preliminary tests were carried out using either pure hydrogen or formic acid (2.3% He diluted) as a hydrogen source using either Re/G or ReO_*x*_/G as a catalyst in a continuous reactor heated by conventional heating. The reaction was carried out at 320 °C. Also, a test using 285 mg ReO_*x*_/G and 2 g of commercial Fe_3_O_4_ was performed using conventional heating. Alternatively, magnetic induction was used under the same conditions and a catalytic bed composed of a physical mixture of 285 mg of ReO_*x*_/G and 2 g of magnetic nanoparticles (MNPs). The reactor was a high-frequency coil working at 100 kHz (IDPartner) that allows controlling the magnetic field amplitude between 0 and 42 mT. The catalyst (with or without MNPs) was loaded in the reactor and the reaction temperature was monitored by a thermocouple placed in the middle of the catalytic bed. Control experiments confirmed that the thermocouple did not influence the induction heating. Also, prior to testing, a series of blank tests were performed with the MNPs: the commercial MNPs without the catalyst were placed in both a conventional reactor and a magnetic reactor. A blank test was also performed using only SiC in the conventional reactor.

The reaction products and reactants were analyzed online using a gas chromatograph (Agilent 8860) equipped with a flame ionization detector (FID) with a DB-Fatwax capillary column and a thermal conductivity detector (TCD) with a Carboxen 1010 column. To identify the reaction products a mass spectrometer (Agilent 5977B GC/MSD) connected to the chromatograph was used.

Anisole conversion and product selectivity are calculated as follows:
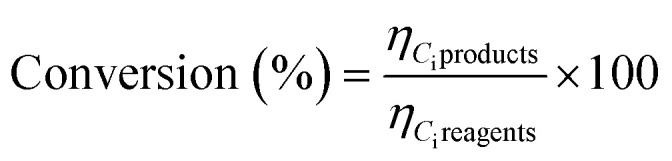

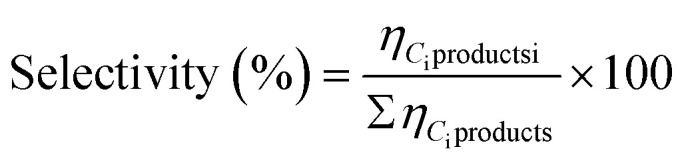
The carbon balance was better than 95%, estimated by comparing the calibrated GC peak areas of the blank anisole with the sum of unreacted anisole and detected products. For the HDO with FA, the estimation included the carbon introduced by FA and the carbon contained in CO and CO_2_ formed during the reaction.

## 3. Results and discussion

### 3.1 Characterization results

The X-ray diffraction patterns of the bare support and the catalysts in Fig. S1 are dominated by the diffraction at 2*θ* of 26.3°, which is characteristic of highly structured graphitic carbon (002) (graphite, JCPDS#01-075-1621). For the sample ReO_*x*_/G, a small peak can also be observed at 37.3° which corresponds to ReO_2_ (JCPDS#24-1009). This may indicate that ReO_3_ is found as larger particles than Re(0).

**Fig. 1:**
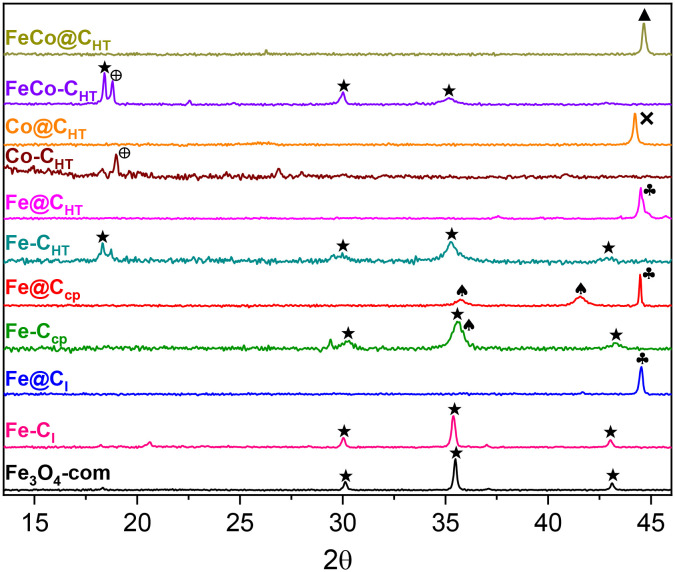
XRD patterns of the encapsulated MNPs before and after heat treatment (800 °C, 3 h, He) and commercial magnetite. ★Fe_3_O_4_, ♠FeO 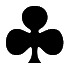
Fe(0), ⊕Co(OH)_2_
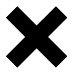
Co(0), ▲FeCo.

Regarding the XRD analysis of the magnetic nanoparticles, measurements were conducted before and after thermal treatment, with the results presented in Fig. 1. The non-encapsulated commercial sample, Fe_3_O_4_, exhibited peaks at 30.1°, 35.5°, and 43.1° characteristic of Fe_3_O_4_ (JCPDS#26-1136), corresponding to the (220), (311) and (400) planes of inverse cubic spinel magnetite. Meanwhile, Fe–C_I_ displayed a similar pattern compared to Fe_3_O_4_; the diffractions of Fe–C_CP_, Fe–C_HT_ and FeCo–C_HT_ are slightly wider. These peaks confirm the presence of Fe_3_O_4_, already formed during hydrothermal synthesis in the autoclave. For Co–C_HT_, the pattern exhibits a single peak at 18.1° corresponding to the (001) plane of Co(OH)_2_ (JCPDS#46-0605). This compound may be formed during the initial stages of the hydrothermal process, which possibly occurs due to the hydrolysis of the homogeneous aqueous solution of cobalt nitrate.^48^ This peak is also present in FeCo–C_HT_. For the Fe-based heat-treated samples, Fe@C_I_, Fe@C_CP_ and Fe@C_HT_, a single peak is observed at 44.5°, corresponding to the (110) plane of metallic Fe (JCPDS#06-0696).^49^ Additionally, Fe@C_CP_ exhibits two peaks at 35.7° and 41.6° ascribed to FeO (JCPDS#01-074-1886).^50^ In the case of Co@C_HT_, the presence of metallic cobalt is confirmed by the peak at 44.2° due to the (111) plane (JCPDS#05-0727).^51^ For FeCo@C_HT_, a single peak at 44.6° is found, which for this sample can be ascribed to the (110) plane of an iron–cobalt alloy.^52^ Moreover, the characteristic diffraction of graphite at 26.3° is not observed on the patterns. The absence of this peak was expected as the thermal treatment led to the formation of a thin layer of graphite, as will be verified by microscopy. The XRD patterns demonstrated that during the pyrolysis at 800 °C, the metal oxides were reduced to metal nanoparticles.^53^

**Fig. 2:**
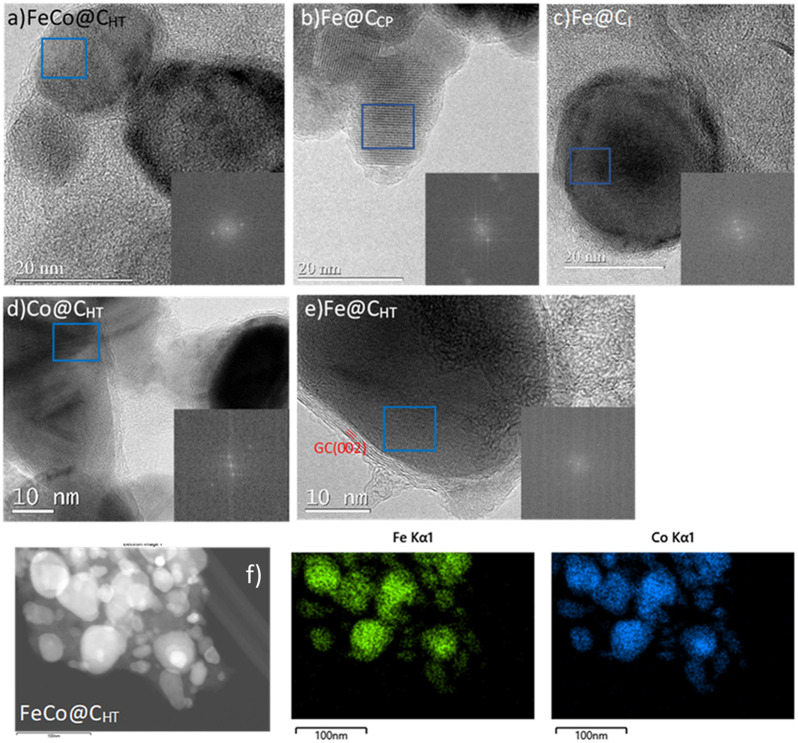
Selected TEM images of (a) FeCo@C_HT_, (b) Fe@C_CP_, (c) Fe@C_I_, (d) Co@C_HT_, and (e) Fe@C_HT_ magnetic nanoparticles. HAADF-STEM images of (f) FeCo@C_HT_ and EDS mapping of Fe (green) and Co (blue) in FeCo@C_HT_.

Fig. 2a–e display selected TEM images of all the magnetic materials, and Fig. 2f shows STEM-EDS images along with the elemental mapping of FeCo@C_HT_. The images reveal the formation of quasi-spherical nanoparticles. Furthermore, all the samples exhibit a well-defined carbon layer surrounding the particles, with the exception of Fe@C_CP_. This phase shows an interplanar spacing of 0.34 nm corresponding to the *d*-spacing of the (002) plane of graphitic carbon, and its thickness is between 3 and 8 nm. Fe@C_I_ graphitic coating is formed by fewer carbon layers. In contrast, Fe@C_CP_ displays a carbon coating environment, but it does not show the formation of a graphitic layer.

The elemental mapping of FeCo@C_HT_, also in Fig. 2, which shows a homogeneous distribution of Fe and Co, confirms the formation of a FeCo alloy throughout the materials as XRD indicated. The particle size of the MNPs was in the range of 20–40 nm. Selected-area electron diffraction (SAED) patterns of the MNPs showed interplanar spacings, in good agreement with XRD. Reflections at *d* ≈0.20 nm in Fe@C_I_ and Fe@C_HT_ matched the (110) plane of metallic Fe, while *d* ≈0.22 nm in Co@C_HT_ corresponded to the (100) plane of metallic Co. For FeCo@C_HT_, the spacing of *d* ≈0.20 nm was assigned to the (110) plane of the FeCo alloy, consistent with the EDS analysis. In Fe@C_CP_, a spacing of *d* ≈0.28 was observed, characteristic of Fe_3_O_4_. These results confirm the strong correlation between SAED and XRD analyses.

**Fig. 3:**
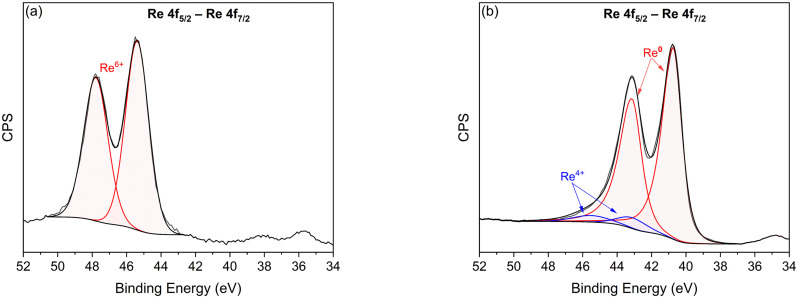
*In situ* XPS of (a) the Re 4f region for the ReO_*x*_/G catalyst and (b) the Re 4f region for the Re/G catalyst.

ReO_*x*_/G and the *in situ* reduced catalyst Re/G were studied by XPS. The Re 4f regions of the Re/G and ReO_*x*_/G catalysts are shown in Fig. 3 and could be decomposed into two components corresponding to the 7/2 and 5/2 orbitals which are split by 2.4 eV.^54^ The 4f region of ReO_*x*_/G in Fig. 3a can be deconvoluted into one component at 45.4 eV and 47.8 eV for the 7/2 orbital and the 5/2 orbital respectively, which correspond to Re^6+^. For the Re/G catalyst in Fig. 3b, the Re 4f_7/2_ region displayed contributions at 40.6 eV and 43.5 eV, which correspond to Re^0^ and Re^4+^.^55^ The relative contribution of Re^0^, 93.3%, confirms that Re is mainly found in the most reduced phase. Regarding the Re/C ratio, for the Re/G catalyst it is 4.0 × 10^−2^ and for ReO_*x*_/G it is 6.7 × 10^−3^, which indicates that rhenium is more dispersed on the support on the Re/G catalyst as also DRX suggested.

XPS was employed to analyze the chemical state of the carbon, iron and cobalt atoms. The C 1s XPS spectra of the materials are included in Fig S2 and could be well fitted into four components. The peak at 284.6 eV is attributed to the C–C bonds of graphitic carbon, which illustrates the delocalized sp^2^-hybridized carbon. The peaks at 285.7–286.2, 288.0–288.5 and 290.5–291.0 eV are attributed to the C–O and C=O bonds and to the π–π* satellite, respectively.^56^ The samples prepared by hydrothermal method show similar binding energies and similar relative atomic percentages of C–C sp^2^, which are included in Table 1. The sample Fe@C_CP_ presents a significant contribution at 289.6 eV corresponding to the presence of carbonates on the surface. The reason for the appearance of this phase is not clear, but it could be due to the higher amount of iron oxides, as the XPS result in Table 1 also shows, which could serve as adsorption sites for CO_2_.^57,58^ Also, the sample Fe@C_I_ displays a higher contribution of C–O species compared to the other materials.

**Table 1:** XPS C 1s, Fe and Co binding energies (eV) of the magnetic nanomaterials

BE, eV (at%)													
Phases	C 1s					Fe 2p_3/2_			Co 2p_3/2_			Fe/C	Co/C
	C–C sp2	C–O	C=O	π–π*	CO_3_^2−^	Fe^0^	Fe^2+^	Fe^3+^	Co^0^	Co^2+^	Co^3+^		
Fe@C_HT_	284.6 (68.0)	285.7 (17.9)	288.0 (6.7)	291.0 (7.4)	—	707.0 (37.2)	710.1 (29.6)	711.5 (33.1)	—	—	—	0.03	—
Co@C_HT_	284.6 (72.7)	286.2 (8.2)	288.3 (8.2)	290.5 (10.9)	—	—	—	—	778.2 (60.2)	780.7 (18.9)	782.3 (20.8)	—	0.07
FeCo@C_HT_	284.6 (69.1)	286.2 (12.9)	288.3 (11.4)	290.8 (6.6)	—	707.0 (26.2)	710.2 (42.0)	711.3 (31.8)	778.5 (42.9)	780.8 (25.1)	782.3 (32.0)	0.08	0.06
Fe@C_CP_	284.6 (51.8)	286.2 (6.7)	288.5 (16.9)	291.0 (2.5)	289.6 (22.2)	—	710.3 (47.9)	711.6 (52.1)	—	—	—	0.12	—
Fe@C_I_	284.6 (51.0)	285.9 (34.0)	288.5 (11.0)	290.5 (4.0)	—	707.0 (7.7)	710.5 (45.9)	711.8 (46.4)	—	—	—	0.08	—

**Fig. 4:**
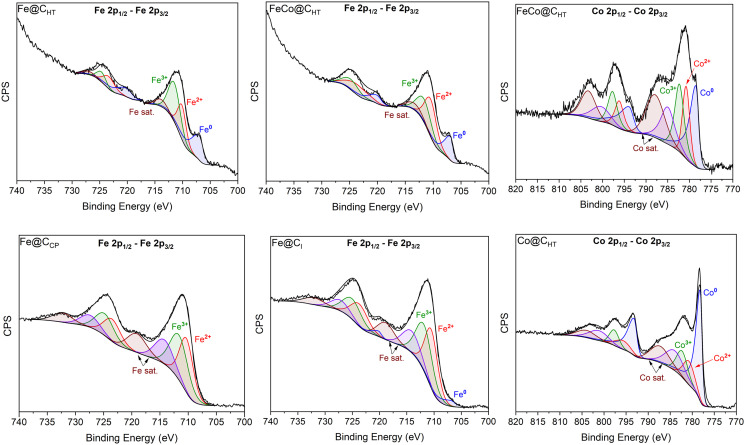
XPS of the Fe 2p and Co 2p region for the Fe@C_HT_, FeCo@C_HT_, Fe@C_CP_, Fe@C_I_ and Co@C_HT_ MNPs.

The Fe 2p 3/2 and Co 2p 3/2 regions are shown in Fig. 4, and Table 2 summarizes the binding energies (BEs) and relative proportions for all the catalysts. As shown in Fig. 4, the Fe 2p_3/2_ region for Fe@C_HT_, FeCo@C_HT_, Fe@C_I_ and Fe@C_CP_ could be deconvoluted into two components with binding energies at 711.8–711.3 eV and 710.5–710.1 eV, which correspond to Fe(iii) and Fe(ii), respectively, in the Fe–O bond. In addition, Fe@C_HT_, FeCo@C_HT_ and Fe@C_I_ also display a component at 707 eV due to metallic Fe.^59^ As included in Table 1, the relative contribution of Fe(0) is 37.2%, 23.4% and 7.7% in Fe@C_HT_, FeCo@C_HT_ and Fe@C_I_, respectively. Concerning the Co 2p_3/2_ region, shown in Fig. 4, for Co@C_HT_ and FeCo@C_HT_, it can be deconvoluted into three components with binding energies at 782.3, 780.7–780.8 and 778.2–778.5 eV, which can be attributed to Co(ii), Co(iii), and metallic Co, respectively.^60^ The relative contribution of Co(0) is 60.2% and 42.9% in Co@C_HT_ and FeCo@C_HT_. The BE values obtained in the Fe 2p and Co 2p regions for the FeCo alloy agree with the values reported in the literature.^61–63^

**Table 2:** Coercivity (*H*_c_) and saturation magnetization (*M*_s_) of MNPs

	*H*_c_ (Oe)	*M*_s_ (emu g^−1^)
Fe@C_HT_	228	164
Co@C_HT_	183	94
FeCo@C_HT_	44	226
Fe@C_CP_	119	7
Fe@C_I_	160	58
Fe_3_O_4_	97	82

The discrepancies between XRD and XPS can be explained by considering that XPS is a surface-sensitive technique and that the graphite layer over the MNPs is 3–8 nm in depth. Hence, the results obtained for the Fe 2p and Co 2p regions arise mainly from oxidized species that have remained unencapsulated and from the outermost Fe and Co layers of the nanoparticles which can be partially oxidized. For the sample Fe@C_I_, the low percentage of Fe(0) may be due to the lesser extent of encapsulation. Similarly, although the XRD of Fe@C_CP_ shows a metallic iron phase, the lack of proper encapsulation results in XPS detecting only the oxide components.

TGA measurements were conducted on Fe@C_HT_, Co@C_HT_, FeCo@C_HT_ Fe@C_I_ and Fe@C_CP_ (Fig. S3). The samples exhibit both weight gain and weight loss, which can be attributed to different processes taking place under the oxidative atmosphere.

Firstly, Fe@C_HT_ and Co@C_HT_ showed a weight gain of 5–10% with a maximum around 400 °C which can be assigned to the oxidation of non-encapsulated Fe and Co metallic phases into oxides. This was followed by a weight loss, much more pronounced for Co@C_HT_, consistent with the combustion of the more reactive, poorly graphitized carbon shell, likely accelerated by the catalytic effect of Co. FeCo@C_HT_ shows a pronounced and sustained weight gain starting above 500 °C, suggesting extensive oxidation of the Fe-Co alloy core, with carbon combustion playing only a minor role. Fe@C_I_ only showed a weight gain starting at temperatures above 500 °C. This may suggest either a more stable carbon layer or a higher encapsulation degree of the metallic phases. In contrast, only Fe@C_CP_ exhibited weight loss: after an initial decrease below 200 °C, two steps centered at ∼250 and ∼300 °C are observed, attributed to oxidation of graphitic and amorphous carbon,^46^ in agreement with microscopy observations. The absence of weight gain for this sample is consistent with XPS results showing no detectable metallic Fe; once Fe is already oxidized, no further oxidation can be registered by TGA. Overall, the results indicate that carbon reactivity follows the trend C_HT_ > C_CP_ > C_I_, while the extent of core oxidation is most pronounced for FeCo@CHT and Fe@C_I_. The different temperatures and magnitudes of these processes reflect the varying encapsulation efficiency and the intrinsic stability of the carbon supports employed. To summarize, the weight changes can be ascribed to the transformation of the unencapsulated iron/cobalt phases under the oxidative atmosphere and/or to the decomposition of the carbon layer.^46^

**Fig. 5:**
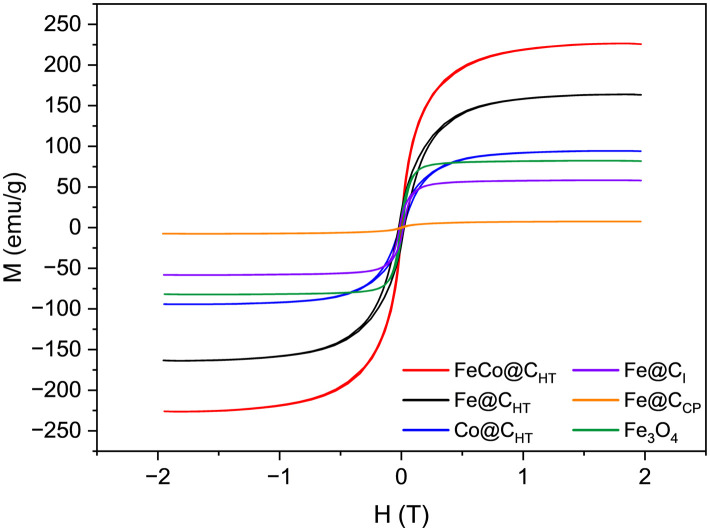
Hysteresis loop measured by VSM on the MNPs and Fe_3_O_4_ at 300 K.

The magnetic properties of Fe@C_HT_, FeCo@C_HT_, Co@C_HT_, Fe@C_I_ and Fe@C_CP_ as well as commercial magnetite (Fe_3_O_4_) nanoparticles were measured using vibrating sample magnetometry (VSM). The saturation magnetization (*M*_s_) and coercivity (*H*_c_) were determined from the hysteresis loops, shown in Fig. 5, at 300 K and the values are compiled in Table 2. All materials have saturation magnetization values that correspond to those typical of ferromagnetic materials.^25,30^ Ferromagnetic particles exhibit distinctive magnetization (*M*/*H*) profiles that allow for the conversion of radiofrequency into heat through hysteresis losses. Among the materials, those MNPs prepared by the HT method presented the most interesting magnetic properties for their use in heterogeneous catalysis. Specifically, FeCo@C_HT_ showed the highest saturation magnetization and the lowest coercivity among the studied samples, with a saturation value close to those previously reported in the literature (233–237 emu g^−1^).^52^ In contrast, Co@C_HT_ exhibited the lowest saturation and highest coercivity.

Notably, although the graphite-encapsulated iron-based materials form metallic iron, they exhibit variations in both saturation magnetization and coercivity. As mentioned in the experimental section, the hydrothermal synthesis was a modification of Lee *et al.*'s^46^ method. They achieved graphite encapsulation of Fe and Co MNPs with *M*_s_ values of 86.6 and 43.8 emu g^−1^, respectively. In our research, the MNPs Fe@C_HT_ and Co@C_HT_ exhibited *M*_s_ values of 164 and 94 emu g^−1^, which are greater than those found in the original study. These results demonstrate that the revised synthesis technique produces MNPs with improved magnetic characteristics, making them suitable for applications in catalytic magnetic induction. Among the tested samples, Fe@C_CP_ is the material with the lowest *M*_s_, which can be associated to the higher percentage of carbon in this sample, related to the TGA mass loss (9.7%).

### 3.2 Catalytic performance

In this work, rhenium-based catalysts were used for the hydrodeoxygenation reaction of anisole with formic acid in the vapor phase at ambient pressure and 320 °C, employing conventional heating and magnetic induction heating.

**Scheme 1:**
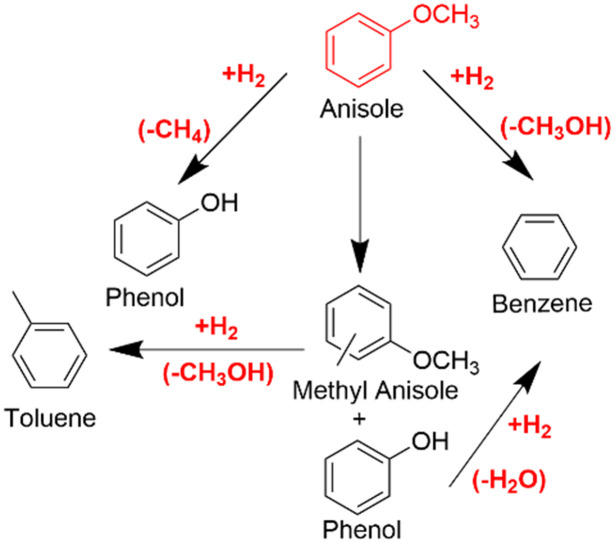
Possible reaction pathways for anisole conversion for HDO reactions. Reactions are postulated on the basis of the products identified.

The hydrogenation of anisole can follow multiple pathways, as shown in Scheme 1. During the hydrogenation of the –OCH_3_ group, the O–CH_3_ bond may break, leading to the formation of phenol and methane, *i.e.* hydrogenolysis, or alternatively, cleavage may occur at the oxygen–aromatic bond, resulting in methanol and benzene, *i.e.* demethoxylation (DMO). Without the intervention of hydrogen, intermolecular alkylation can occur, forming phenol and methyl anisole, which can react with H_2_ to form toluene.^64,65^

Fig. S4 shows the preliminary results using a conventional heating setup and Re/G or ReO_*x*_/G as a catalyst, for the latter evaluating both H_2_ and FA as hydrogen sources. In all the reaction tests, an initial pronounced deactivation of the catalyst occurs until 150 min, where the conversion and selectivity to benzene decreased. This stage was followed by a more stable conversion and selectivity profile, although conversion seems to smoothly decay. From this time, both catalysts show a similar conversion, 5–6%, and the selectivity was directed towards benzene, *ca.* 65–70%, and phenol, *ca.* 28–32%, while toluene and methyl anisole (data not shown) were only produced with a selectivity below 5%. Also, the results of conversion and selectivity obtained using ReO_*x*_/G and H_2_ or FA as hydrogen sources are similar. An additional test was conducted to study the conversion and selectivity obtained with the physical mixture of ReO_*x*_/G and commercial Fe_3_O_4_ using conventional heating. A notable increase in conversion up to 14% was observed as well as a change in selectivity, yielding 35% benzene and 65% phenol. This indicates that the presence of iron oxides is negative for the efficiency of the hydrodeoxygenation reaction, likely due to the lower activity of iron oxides in breaking C–O bonds.^66^ In this reaction, iron acts as a promoter when combined with supported rhenium oxides, which explains the higher conversion.^67^ Anisole HDO has been previously evaluated using Re-based catalysts. However, data about their use in gas-phase HDO are scarce. Rezaei *et al.*^67^ evaluated FeReO_*x*_/ZrO_2_ catalysts in the HDO of anisole in the gas phase at 350 °C and WHSV of 19.9 h^−1^ and observed a selectivity to benzene of 43.3% and only 4.4% selectivity to toluene. The initial deactivation observed may be due to strongly adsorbed anisole and formic acid molecules occupying the available active sites, as evidenced by the post-reaction TPD experiment (Fig. S5), where signals corresponding to the characteristic masses of the mentioned molecules were detected, indicating surface blockage.

Besides Re, Mo catalysts have been evaluated in the gas phase, also taking advantage of the variety of its oxidation states. Shetty *et al.*^65^ reported that toluene was a minor product in the HDO of anisole over MoO_3_/ZrO_2_, obtained *via* a secondary reaction pathway from *m*-cresol intermediates. In the reaction at 320 °C, they obtained benzene as the main product at 27% and phenol at 23%. The HDO of anisole has been studied using molybdenum carbides at low temperatures (150–250 °C), using kinetic conversions below 15%, achieving a benzene selectivity of 80–90%.^12,68^ More literature reports were found related to the use of Re-based catalysts for HDO of anisole in the liquid phase. For example, Ghampson *et al.*^69^ investigated ReO_*x*_/CNF catalysts in liquid-phase HDO at 300 °C, showing that Re species of the type Re^6+^ promote direct C–O bond cleavage. Similarly, Wang *et al.*^70^ examined Cu–ReO_*x*_/SiO_2_ catalysts at 320 °C in the liquid phase, finding that the Cu–Re interaction enhanced anisole adsorption while facilitating benzene desorption, resulting in 50% BTX yield at optimal Cu/Re ratios. Despite the key role of the solvent in the liquid phase reactions, the reported literature has shown that rhenium provides several oxidation states, *i.e.*, Re^7+^, Re^6+^, and Re^4+^, that can facilitate H_2_ activation and subsequent C–O bond cleavage, even at relatively low temperatures.^71^

For these catalytic systems, the selectivity towards benzene can be attributed to the strong oxophilicity of Re species, which may favor the hydrogenolysis of both anisole and phenol.^71,72^ Interestingly, the use of formic acid as a hydrogen source does not significantly modify the conversion or the selectivity. This result is in contrast with other studies, where formic acid outperforms molecular H_2_ in other HDO reactions such as benzylic ketones using Pd/HPC-NH_2_ as a catalyst.^73^ This improvement was also observed in the hydrogenation and HDO of lignin-derived monomers, including guaiacol and syringol, yielding various aromatics with a lower oxygen weight content compared to the use of external H_2_. Nevertheless, those reactions were performed in the liquid phase and the reaction mechanism and hydrogen availability would differ from that of our system. In our previous research on the gas phase hydrogenation of crotonaldehyde using Cu and Re on a graphite support,^74^ we found that the type of hydrogen source (formic acid or molecular hydrogen) significantly impacts both activity and selectivity. Specifically, catalysts based on Re, which showed no activity with H_2_, became active when formic acid was utilized. This indicates that the enhancing effect of formic acid is not the same for all systems but depends on various factors, including the reaction phase and the interactions between the substrate and the surface.

ReO_*x*_/G was selected as the catalyst to further evaluate the use of magnetic heating employing FA as the hydrogen source. Hence, the same reaction was carried out at 320 °C using magnetic induction and varying the MNPs. When the reactor was placed inside the coil a magnetic field was applied to reach the target temperature of 320 °C.

**Fig. 6:**
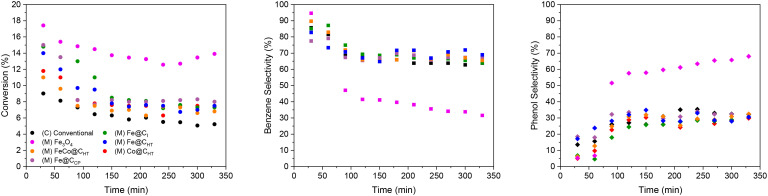
Conversion (circles), selectivity to benzene (squares) and phenol (diamonds) as a function of time during conventional (C) and magnetically induced (M) anisole HDO over the ReO_*x*_/G catalyst and MNPs at variable magnetic field amplitudes. Reaction conditions: H_2_ source: FA, 2.7% vol. anisole, 100 kHz, 320 °C.

The results in Fig. 6 show a similar trend in the conversion to that of conventional heating. At the beginning of the reaction the conversion was in the range 11–17% and falls to around 7% when using the encapsulated materials. However, when commercial nanoparticles of Fe_3_O_4_ were used, the conversion of anisole was close to 14% as seen in conventional heating reactions.

Differences are also observed in the selectivity values. While encapsulated nanoparticles reached 65% selectivity to benzene, the unencapsulated Fe_3_O_4_ showed selectivity to benzene of 30%. This again reflects that MNPs, if not encapsulated, can influence conversion and selectivity.

Table 3 summarizes the magnetic fields required to heat different MNPs to the reaction temperature at 100 kHz. Each material exhibits a unique value within the range of 16 to 34 mT. When FeCo@C_HT_ was used, the required field was the lowest, 16 mT. The MNPs based solely on encapsulated iron show comparable values (20, 21 and 23 mT). The Co@C_HT_ nanoparticles required a field of 34 mT, making them the material with the highest field demand. Commercial nanoparticles, requiring a slightly higher field (26 mT), exhibit a notable change in selectivity due to the lack of encapsulation. These slight variations can be attributed to differences observed in XRD analyses, where Fe@C_CP_, after heat treatment, retained traces of iron oxide (FeO).

**Table 3:** Catalytic performance of magnetically induced anisole HDO using various MNPs at 320 °C with ReO_*x*_/G

Heating agent (HA)	Field (mT)	Conv. (%)	S benzene (%)	S phenol (%)	S toluene (%)
Fe@C_HT_	20	7.6	69.3	30.4	<1
Co@C_HT_	34	7.4	67.5	27.9	4.6
FeCo@C_HT_	16	7.0	67.1	30.1	2.8
Fe@C_CP_	23	8.1	67.4	31.8	<1
Fe@C_I_	21	8.1	67.0	27.4	5.5
Fe_3_O_4_	26	16.4	34.7	63.1	<1
Methylanisole selectivity <1					

The decomposition of formic acid was evaluated using ReO_*x*_/G as a catalyst and Fe_3_O_4_ since iron has been reported to be active on the dehydration of FA, resulting in CO + H_2_O.^75^ The results for conversion and CO_2_ selectivity are presented in Fig. S6 and indicate thermal decomposition as the main path under these conditions. Formic acid conversion at 320 °C reached a value of 100% in all three reactions including a previous blank test. The selectivity towards CO_2_ was 80–84% with conventional heating and 76% with magnetic heating. Therefore, the CO values obtained in the catalytic system were in the range of (0.8–1.4) × 10^−5^ mol min^−1^, corresponding to approximately 16–24% of the formic acid decomposition pathway. With these results, H_2_ can be supplied in excess for the hydrodeoxygenation reaction of anisole, with H_2_/anisole ratios of 5.0–4.6 (Table S1). The lower selectivity to CO_2_ obtained with magnetic heating could be attributed to the presence of Fe_3_O_4_.

In the HDO of anisole performed under magnetic heating with Fe_3_O_4_ as a heating agent, a full conversion was achieved without losing CO_2_ selectivity of 80%, complemented by a CO formation rate of 1.06 × 10^−5^ mol min^−1^ and a H_2_/anisole ratio of 4.8. The same trend was observed for FeCo@C_HT_, where complete conversion was measured, with a CO_2_ selectivity of 90%. The latter gave the lowest CO produced (5.29 × 10^−6^ mol min^−1^) and highest H_2_/anisole ratio (5.4), marking the positively significant impact of FeCo encapsulation to reduce dehydration of formic acid and promote generation of H_2_ for the HDO reaction.

We have performed TXRF and XPS analyses of the spent catalyst ReO_*x*_/G after the HDO reaction, included in Table S2. The TXRF measured values are consistent with the nominal Re loading, and the Re/C values of the spent catalyst after reaction under H_2_ or FA are similar to that of the fresh catalyst, confirming that no leaching occurred.

To check the stability of the MNPs, XRD analysis (Fig. S7) of the used MNPs in the catalytic experiments was performed. All the MNPs showed diffractions at 44.5°, ascribed to Fe(0) and/or at 44.2°, attributed to Co(0). Moreover, the materials FeCo@C_HT_, Fe@C_CP_, Fe@C_I_, and Fe_3_O_4_ exhibited diffractions at 30°, 35.5°, 37°, and 43.1°, which are attributed to Fe_3_O_4_, indicating that in the encapsulated MNP, oxidation of metallic iron has occurred during the reaction. In Co@C_HT_, metallic cobalt remains as the only observable phase. In fact, a metallic phase is observed in all the compounds, which indicates that the encapsulation helped to maintain the structure of the MNPs throughout the reaction, since complete oxidation of the material has not occurred during the reaction time, which would lead to a change in its magnetic properties and a change in the magnetic field necessary to reach 320 °C. This aligns with earlier studies that emphasize the significance of thermal stability in catalysis and the role of graphite encapsulation.^31,35^ In these studies, magnetic non-encapsulated nanoparticles have been observed to experience sintering and loss of effectiveness when subjected to high temperatures for long periods. To address this limitation, Martínez-Prieto *et al.*^31^ developed FeCo and Co nanoparticles encapsulated in carbon (FeCo@C and Co@C), which were capable of sustaining high temperatures (>600 °C) during gas-phase reactions such as CO_2_ methanation, propane dehydrogenation and dry reforming. These carbon-encapsulated MNPs retained their morphology and heating performance over time. Similarly, Cerezo-Navarrete *et al.*^35^ reported the synthesis of FeCo@Ni nanoparticles *via* an organometallic approach in mesitylene. Although effective under magnetically induced catalytic conditions for the conditions for the HDO of vanillin in water (conversion of 35%), those materials required carbon encapsulation to mitigate deactivation due to thermal sintering and magnetic agglomeration during prolonged exposure to high-frequency alternating magnetic fields (50–100 mT at 300 kHz). Finally, the carbon-coated MNPs demonstrated recyclability and stability and they achieved over 90% conversion. Magnetic induction heating has also been applied in different biomass valorization reactions, particularly in hydrogenations and HDO processes in the liquid phase (typically at 30–100 mT at 300 kHz). Asensio *et al.*^20^ demonstrated that MNPs are linked to the catalytic phase, which can create small hot areas that go beyond the boiling point of the solvent, allowing full HDO of acetophenone, furfural, and HMF under 3 bar of H_2_. The reaction took place under less severe conditions than those required for heterogeneous catalysis. This shows the potential of magnetically induced “nanoreactors” to facilitate demanding transformations through rapid, localized, and efficient heating. Crucially, the intimate contact between the heating agent (Fe_2.2_C) and the active phase (Ru) was essential to achieve high selectivity and avoid an undesired hydrogenation of the aromatic ring. In addition to the above, in these studies the synthesis of MNPs reported in the literature is usually more complex, since it requires organometallic precursors. For instance, Mustieles Marin *et al.*^36^ used {Fe[N(SiMe_3_)_2_]_2_}_2_ and Ni[iPrNC(CH_3_)NiPr]_2_ as precursors for the formation of nanoparticles with a FeNi core in the HDO of vanillin (47 mT at 100 kHz). However, its application in gas-phase systems remains less explored. Regarding gas-phase reactions, magnetic induction has also been used in processes such as CO_2_ hydrogenation,^26,76^ although these studies are still less common. In our work, we provide a new approach to the application of magnetic induction, demonstrating their efficiency in catalytic gas phase reactions involving biomass-derived molecules.

## 4. Conclusions

Characterization revealed that the MNPs formed by a thermal method showed variable carbon coverage depending on the carbon source used, with glucose being the most effective in promoting the formation of a graphitic layer. Encapsulation was not complete in all cases, as some materials remained partially coated or formed oxides. Thermal treatments allowed the *in situ* reduction of metal oxides to metallic nanoparticles. MNPs synthesized by the hydrothermal method exhibited the best magnetic properties for magnetic induction catalysis. In particular, FeCo@C_HT_ showed the highest saturation magnetization and the lowest coercivity, making it the most suitable material for this purpose. The presence of a graphite layer contributed to the thermal stability and oxidation resistance of the nanoparticles during the reaction. Additionally, a higher percentage of metallic iron and cobalt correlated with better encapsulation, enhancing nanoparticle stability, while poor encapsulation favored oxide formation, negatively impacting magnetic properties. Regarding the catalytic performance, the ReO_*x*_/G catalyst showed an anisole conversion of around 8%, regardless of whether hydrogen or formic acid was used as the hydrogen source. The use of magnetic induction did not significantly affect the conversion, but commercial Fe_3_O_4_ nanoparticles, when non-encapsulated, achieved a higher conversion of 13%, with a predominant selectivity towards phenol. Materials with graphitic encapsulation, such as FeCo@C_HT_, displayed better magnetic properties and greater stability during the reaction process. In this system, the reaction is slightly to moderately endothermic (+70 to +140 kJ mol^−1^ anisole), requiring continuous external energy input to proceed. Nevertheless, even in exothermic HDO reactions, magnetic heating offers additional advantages, where a rapid and localized energy input facilitates the initiation step. Once the reaction becomes self-sustaining, the magnetic field can be switched off, thereby minimizing the risk of overheating while improving both energy efficiency and operational safety. In summary, the results highlight the importance of selecting the appropriate synthesis method to achieve the desired magnetic properties and effective encapsulation, as these factors significantly influence nanoparticle stability and product selectivity. The combination of rhenium with encapsulated magnetic nanoparticles emerges as a promising strategy to enhance catalytic efficiency in hydrogenation processes, with potential applications in the conversion of anisole and other phenolic compounds. Furthermore, since all samples were subjected to the same thermal treatment, it can be concluded that the hydrothermal process is the most suitable for achieving greater coverage and better carbon crystallinity.

## Author contributions

VNO: investigation, writing – original draft. MD: investigation. GS: conceptualization, review and editing, funding acquisition. ABD: conceptualization, project administration, supervision, writing, review and editing, funding acquisition.

## Conflicts of interest

There are no conflicts to declare.

## Supplementary Material

CY-016-D5CY00707K-s001

## Data Availability

Data for this article were obtained during the PhD thesis of VNO at Universidad Autónoma de Madrid. Other data not included in the SI can be requested from the authors. Supplementary information is available. See DOI: https://doi.org/10.1039/d5cy00707k.

## References

[cit1] Demirbaş A. (2001). Biomass Resource Facilities and Biomass Conversion Processing for Fuels and Chemicals. Energy Convers. Manage..

[cit2] Zabed H., Sahu J. N., Boyce A. N., Faruq G. (2016). Fuel Ethanol Production from Lignocellulosic Biomass: An Overview on Feedstocks and Technological Approaches. Renewable Sustainable Energy Rev..

[cit3] Mankar A. R., Pandey A., Modak A., Pant K. K. (2021). Pretreatment of Lignocellulosic Biomass: A Review on Recent Advances. Bioresour. Technol..

[cit4] Chio C., Sain M., Qin W. (2019). Lignin Utilization: A Review of Lignin Depolymerization from Various Aspects. Renewable Sustainable Energy Rev..

[cit5] Ragauskas A. J., Beckham G. T., Biddy M. J., Chandra R., Chen F., Davis M. F., Davison B. H., Dixon R. A., Gilna P., Keller M., Langan P., Naskar A. K., Saddler J. N., Tschaplinski T. J., Tuskan G. A., Wyman C. E. (2014). Lignin Valorization: Improving Lignin Processing in the Biorefinery. Science.

[cit6] Runnebaum R. C., Nimmanwudipong T., Block D. E., Gates B. C. (2011). Catalytic Conversion of Anisole: Evidence of Oxygen Removal in Reactions with Hydrogen. Catal. Lett..

[cit7] Vu H. P., Nguyen L. N., Vu M. T., Johir M. A. H., McLaughlan R., Nghiem L. D. (2020). A Comprehensive Review on the Framework to Valorise Lignocellulosic Biomass as Biorefinery Feedstocks. Sci. Total Environ..

[cit8] Philippov A., Nesterov N., Pakharukova V., Kozhevnikov I., Martyanov O. (2022). Advanced High-Loaded Ni–Cu Catalysts in Transfer Hydrogenation of Anisole: Unexpected Effect of Cu Addition. Catalysts.

[cit9] Duong N. N., Aruho D., Wang B., Resasco D. E. (2019). Hydrodeoxygenation of Anisole over Different Rh Surfaces. Chin. J. Catal..

[cit10] Dongil A. B., Pastor-Pérez L., Sepúlveda-Escribano A., García R., Escalona N. (2016). Hydrodeoxygenation of Guaiacol: Tuning the Selectivity to Cyclohexene by Introducing Ni Nanoparticles inside Carbon Nanotubes. Fuel.

[cit11] Ruiz P. E., Frederick B. G., De Sisto W. J., Austin R. N., Radovic L. R., Leiva K., García R., Escalona N., Wheeler M. C. (2012). Guaiacol Hydrodeoxygenation on MoS2 Catalysts: Influence of Activated Carbon Supports. Catal. Commun..

[cit12] Lee W. S., Wang Z., Wu R. J., Bhan A. (2014). Selective Vapor-Phase Hydrodeoxygenation of Anisole to Benzene on Molybdenum Carbide Catalysts. J. Catal..

[cit13] Carrales-Alvarado D. H., Dongil A. B., Guerrero-Ruiz A., Rodríguez-Ramos I. (2021). Tandem Catalysts for the Selective Hydrogenation of Butadiene with Hydrogen Generated from the Decomposition of Formic Acid. Chem. Commun..

[cit14] Philippov A. A., Chibiryaev A. M., Martyanov O. N. (2021). Catalyzed Transfer Hydrogenation by 2-Propanol for Highly Selective PAHs Reduction. Catal. Today.

[cit15] Valentini F., Kozell V., Petrucci C., Marrocchi A., Gu Y., Gelman D., Vaccaro L. (2019). Formic Acid, a Biomass-Derived Source of Energy and Hydrogen for Biomass Upgrading. Energy Environ. Sci..

[cit16] Mori K., Futamura Y., Masuda S., Kobayashi H., Yamashita H. (2019). Controlled Release of Hydrogen Isotope Compounds and Tunneling Effect in the Heterogeneously-Catalyzed Formic Acid Dehydrogenation. Nat. Commun..

[cit17] Zhang D., Ye F., Xue T., Guan Y., Wang Y. M. (2014). Transfer Hydrogenation of Phenol on Supported Pd Catalysts Using Formic Acid as an Alternative Hydrogen Source. Catal. Today.

[cit18] Bulushev D. A., Ross J. R. H. (2011). Vapour Phase Hydrogenation of Olefins by Formic Acid over a Pd/C Catalyst. Catal. Today.

[cit19] Elvers B. and Fritz U., Ullmann's Encyclopedia of Industrial Chemistry, Vch, 1990

[cit20] Asensio J. M., Miguel A. B., Fazzini P., van Leeuwen P. W. N. M., Chaudret B. (2019). Hydrodeoxygenation Using Magnetic Induction: High-Temperature Heterogeneous Catalysis in Solution. Angew. Chem..

[cit21] Etemadi H., Plieger P. G. (2020). Magnetic Fluid Hyperthermia Based on Magnetic Nanoparticles: Physical Characteristics, Historical Perspective, Clinical Trials, Technological Challenges, and Recent Advances. Adv. Ther..

[cit22] Díaz-Riascos Z. V., Llaguno-Munive M., Lafuente-Gómez N., Luengo Y., Holmes S., Volatron J., Ibarrola O., Mancilla S., Sarno F., Aguirre J. J., Razafindrakoto S., Southern P., Terán F. J., Keogh A., Salas G., Prina-Mello A., Lacal J. C., del Pozo A., Pankhurst Q. A., Hidalgo M., Gazeau F., Somoza Á., Schwartz S., Abasolo I. (2025). Preclinical Development of Magnetic Nanoparticles for Hyperthermia Treatment of Pancreatic Cancer. ACS Appl. Mater. Interfaces.

[cit23] Palma V., Barba D., Cortese M., Martino M., Renda S., Meloni E. (2020). Microwaves and Heterogeneous Catalysis: A Review on Selected Catalytic Processes. Catalysts.

[cit24] Ortega D. and Pankhurst Q. A., Magnetic Hyperthermia, 2012, 10.1039/9781849734844-00060

[cit25] Wang W., Tuci G., Duong-Viet C., Liu Y., Rossin A., Luconi L., Nhut J. M., Nguyen-Dinh L., Pham-Huu C., Giambastiani G. (2019). Induction Heating: An Enabling Technology for the Heat Management in Catalytic Processes. ACS Catal..

[cit26] Bordet A., Lacroix L.-M., Fazzini P.-F., Carrey J., Soulantica K., Chaudret B. (2016). Magnetically Induced Continuous CO2 Hydrogenation Using Composite Iron Carbide Nanoparticles of Exceptionally High Heating Power. Angew. Chem..

[cit27] Lucia O., Maussion P., Dede E. J., Burdio J. M. (2014). Induction Heating Technology and Its Applications: Past Developments, Current Technology, and Future Challenges. IRE Trans. Ind. Electron..

[cit28] Bordet A., Leitner W., Chaudret B. (2025). Magnetically Induced Catalysis: Definition, Advances, and Potential. Angew. Chem., Int. Ed..

[cit29] Hartwig J., Ceylan S., Kupracz L., Coutable L., Kirschning A. (2013). Heating under High-Frequency Inductive Conditions: Application to the Continuous Synthesis of the Neurolepticum Olanzapine (Zyprexa). Angew. Chem., Int. Ed..

[cit30] Meffre A., Mehdaoui B., Connord V., Carrey J., Fazzini P. F., Lachaize S., Respaud M., Chaudret B. (2015). Complex Nano-Objects Displaying Both Magnetic and Catalytic Properties: A Proof of Concept for Magnetically Induced Heterogeneous Catalysis. Nano Lett..

[cit31] Martínez-Prieto L. M., Marbaix J., Asensio J. M., Cerezo-Navarrete C., Fazzini P. F., Soulantica K., Chaudret B., Corma A. (2020). Ultrastable Magnetic Nanoparticles Encapsulated in Carbon for Magnetically Induced Catalysis. ACS Appl. Nano Mater..

[cit32] Ding H. L., Zhang Y. X., Wang S., Xu J. M., Xu S. C., Li G. H. (2012). Fe3O4@SiO2 Core/Shell Nanoparticles: The Silica Coating Regulations with a Single Core for Different Core Sizes and Shell Thicknesses. Chem. Mater..

[cit33] Lin S. H., Hetaba W., Chaudret B., Leitner W., Bordet A. (2022). Copper-Decorated Iron Carbide Nanoparticles Heated by Magnetic Induction as Adaptive Multifunctional Catalysts for the Selective Hydrodeoxygenation of Aldehydes. Adv. Energy Mater..

[cit34] Mazarío J., Mustieles Marin I., Mencia G., Lopes C. W., Varela-Izquierdo V., Agostini G., Fazzini P. F., Ratel-Ramond N., Chaudret B. (2024). NiCo and NiCo Decorated with Ru Nanoparticles for Magnetically Induced Hydroprocessing of Lignin Models. ACS Appl. Nano Mater..

[cit35] Cerezo-Navarrete C., Marin I. M., García-Miquel H., Corma A., Chaudret B., Martínez-Prieto L. M. (2022). Magnetically Induced Catalytic Reduction of Biomass-Derived Oxygenated Compounds in Water. ACS Catal..

[cit36] Marin I. Mustieles, De Masi D., Lacroix L. M., Fazzini P. F., van Leeuwen P. W. N. M., Asensio J. M., Chaudret B. (2021). Hydrodeoxygenation and Hydrogenolysis of Biomass-Based Materials Using FeNi Catalysts and Magnetic Induction. Green Chem..

[cit37] Luo J., Liang C. (2024). Rhenium in Heterogeneous Catalysis: A Rising Star for Hydrogenation Reactions. ACS Catal..

[cit38] Di X., Shao Z., Li C., Li W., Liang C. (2015). Hydrogenation of Succinic Acid over Supported Rhenium Catalysts Prepared by the Microwave-Assisted Thermolytic Method. Catal. Sci. Technol..

[cit39] Blanco E., Dongil A. B., Ghampson I. T., García-fierro J. L., Escalona N. (2021). Optimizing the Carburization Conditions of Supported Rhenium Carbide for Guaiacol Conversion. Appl. Catal., A.

[cit40] Falcone D. D., Hack J. H., Davis R. J. (2016). Aqueous-Phase Hydrogenation of Saturated and Unsaturated Ketones and Aldehydes over Supported Platinum-Rhenium Catalysts. ChemCatChem.

[cit41] Martínez N., García R., Fierro J. L. G., Wheeler C., Austin R. N., Gallagher J. R., Miller J. T., Krause T. R., Escalona N., Sepúlveda C. (2016). Effect of Cu Addition as a Promoter on Re/SiO2 Catalysts in the Hydrodeoxygenation of 2-Methoxyphenol as a Model Bio Oil Compound. Fuel.

[cit42] Leiva K., Garcia R., Sepulveda C., Laurenti D., Geantet C., Vrinat M., Garcia-Fierro J. L., Escalona N. (2017). Conversion of Guaiacol over Supported ReOx Catalysts: Support and Metal Loading Effect. Catal. Today.

[cit43] Toledo F., Ghampson I. T., Sepúlveda C., García R., Fierro J. L. G., Videla A., Serpell R., Escalona N. (2019). Effect of Re Content and Support in the Liquid Phase Conversion of Furfural to Furfuryl Alcohol and 2-Methyl Furan over ReOx Catalysts. Fuel.

[cit44] Lam E., Luong J. H. T. (2014). Carbon Materials as Catalyst Supports and Catalysts in the Transformation of Biomass to Fuels and Chemicals. ACS Catal..

[cit45] Yang Y., Chiang K., Burke N. (2011). Porous Carbon-Supported Catalysts for Energy and Environmental Applications: A Short Review. Catal. Today.

[cit46] Lee S. J., Jung J., Kim M. A., Kim Y. R., Park J. K. (2012). Synthesis of Highly Stable Graphite-Encapsulated Metal (Fe, Co, and Ni) Nanoparticles. J. Mater. Sci..

[cit47] Bee A., Massart R., Neveu S. (1995). Synthesis of Very Fine Maghemite Particles. J. Magn. Magn. Mater..

[cit48] Pillai A. S., Rajagopalan R., Amruthalakshmi A., Joseph J., Ajay A., Shakir I., Nair S. V., Balakrishnan A. (2015). Mesoscopic Architectures of Co(OH)2 Spheres with an Extended Array of Microporous Threads as Pseudocapacitor Electrode Materials. Colloids Surf., A.

[cit49] Murugesan S., Kuznetsov O., Zhou Z., Khabashesku V. (2019). Fluorescent Superparamagnetic Core-Shell Nanostructures: Facile Synthesis of Fe@C-CNx Particles for Reusable Photocatalysts. Adv. Nanopart..

[cit50] Jiang J., Wen C., Tian Z., Wang Y., Zhai Y., Chen L., Li Y., Liu Q., Wang C., Ma L. (2020). Manganese-Promoted Fe3O4 Microsphere for Efficient Conversion of CO2 to Light Olefins. Ind. Eng. Chem. Res..

[cit51] Deori K., Deka S. (2013). Morphology Oriented Surfactant Dependent CoO and Reaction Time Dependent Co3O4 Nanocrystals from Single Synthesis Method and Their Optical and Magnetic Properties. CrystEngComm.

[cit52] Castrillón M., Mayoral A., Urtizberea A., Marquina C., Irusta S., Meier J. G., Santamaría J. (2013). Synthesis and Magnetic Behavior of Ultra-Small Bimetallic FeCo/Graphite Nanoparticles. Nanotechnology.

[cit53] Gao C., Lyu F., Yin Y. (2020). Encapsulated Metal Nanoparticles for Catalysis. Chem. Rev..

[cit54] Moulder J. F. and Chastain J., Handbook of X-Ray Photoelectron Spectroscopy: A Reference Book of Standard Spectra for Identification and Interpretation of XPS Data, Perkin-Elmer, 1992

[cit55] Wagner C. D. , NIST X-Ray Photoelectron Spectrometry Database, NIST Standard Reference Database 20, Version 4.1, 1991, pp. 1–76

[cit56] Wu F., Huang R., Mu D., Wu B., Chen Y. (2016). Controlled Synthesis of Graphitic Carbon-Encapsulated α-Fe2O3 Nanocomposite via Low-Temperature Catalytic Graphitization of Biomass and Its Lithium Storage Property. Electrochim. Acta.

[cit57] Fabozzi A., Cerciello F., Senneca O. (2024). Reduction of Iron Oxides for CO2 Capture Materials. Energies.

[cit58] Mora Mendoza E. Y., Sarmiento Santos A., Vera López E., Drozd V., Durygin A., Chen J., Saxena S. K. (2019). Iron Oxides as Efficient Sorbents for CO2 Capture. J. Mater. Res. Technol..

[cit59] Li L., Ma P., Hussain S., Jia L., Lin D., Yin X., Lin Y., Cheng Z., Wang L. (2019). FeS2/Carbon Hybrids on Carbon Cloth: A Highly Efficient and Stable Counter Electrode for Dye-Sensitized Solar Cells. Sustainable Energy Fuels.

[cit60] Biesinger M. C., Payne B. P., Grosvenor A. P., Lau L. W. M., Gerson A. R., Smart R. S. C. (2011). Resolving Surface Chemical States in XPS Analysis of First Row Transition Metals, Oxides and Hydroxides: Cr, Mn, Fe, Co and Ni. Appl. Surf. Sci..

[cit61] Zhu D., Chen M., Huang Y., Li R., Huang T., ji Cao J., Shen Z., Lee S. C. (2022). FeCo Alloy Encased in Nitrogen-Doped Carbon for Efficient Formaldehyde Removal: Preparation, Electronic Structure, and d-Band Center Tailoring. J. Hazard. Mater..

[cit62] Yan J., Huang Y., Liu P., Wei C. (2017). Large-Scale Controlled Synthesis of Magnetic FeCo Alloy with Different Morphologies and Their High Performance of Electromagnetic Wave Absorption. J. Mater. Sci.: Mater. Electron..

[cit63] Yang B., Wu Y., Li X., Yu R. (2018). Chemical Synthesis of High-Stable Amorphous FeCo Nanoalloys with Good Magnetic Properties. Nanomaterials.

[cit64] Thompson S. T., Lamb H. H. (2018). Vapor-Phase Hydrodeoxygenation of Guaiacol over Carbon-Supported Pd, Re and PdRe Catalysts. Appl. Catal., A.

[cit65] Shetty M., Anderson E. M., Green W. H., Román-Leshkov Y. (2019). Kinetic Analysis and Reaction Mechanism for Anisole Conversion over Zirconia-Supported Molybdenum Oxide. J. Catal..

[cit66] Liu X., Shen H., Li H. (2024). Functional Catalysts for Self-Supported Hydrodeoxygenation of Anisole in Water. ACS Sustainable Chem. Eng..

[cit67] Sirous-Rezaei P., Jae J., Ha J. M., Ko C. H., Kim J. M., Jeon J. K., Park Y. K. (2018). Mild Hydrodeoxygenation of Phenolic Lignin Model Compounds over a FeReOx/ZrO2 Catalyst: Zirconia and Rhenium Oxide as Efficient Dehydration Promoters. Green Chem..

[cit68] Lu Q., Chen C. J., Luc W., Chen J. G., Bhan A., Jiao F. (2016). Ordered Mesoporous Metal Carbides with Enhanced Anisole Hydrodeoxygenation Selectivity. ACS Catal..

[cit69] Ghampson I. T., Sepúlveda C., García R. A., García L., Escalona N. (2016). Carbon Nanofiber-Supported ReOx Catalysts for the Hydrodeoxygenation of Lignin-Derived Compounds. Catal. Sci. Technol..

[cit70] Wang X., Zhou W., Wang Y., Huang S., Zhao Y., Wang S., Ma X. (2021). Synergistic Effect for Selective Hydrodeoxygenation of Anisole over Cu-ReOx/SiO2. Catal. Today.

[cit71] Blanco E., Cabeza P., Naharro Ovejero V., Contreras C., Dongil A. B., Ghampson I. T., Escalona N. (2023). Effect of Carbon Support and Functionalization on the Synthesis of Rhenium Carbide and Its Use on HDO of Guaiacol. Catal. Today.

[cit72] Ghampson I. T., Pecchi G., Fierro J. L. G., Videla A., Escalona N. (2017). Catalytic Hydrodeoxygenation of Anisole over Re-MoOx/TiO2 and Re-VOx/TiO2 Catalysts. Appl. Catal., B.

[cit73] Ning H., Chen Y., Wang Z., Mao S., Chen Z., Gong Y., Wang Y. (2021). Selective Upgrading of Biomass-Derived Benzylic Ketones by (Formic Acid)–Pd/HPC–NH2 System with High Efficiency under Ambient Conditions. Chem.

[cit74] Naharro-ovejero V., Pascual L., Zarate X., Saavedra-torres M., Schott E. (2025). Gas Phase Hydrogenation of Crotonaldehyde Using Formic Acid as Hydrogen Source over Cu and Re Supported on Graphite. Appl. Catal., B.

[cit75] Gamba O., Noei H., Pavelec J., Bliem R., Schmid M., Diebold U., Stierle A., Parkinson G. S. (2015). Adsorption of Formic Acid on the Fe3O4(001) Surface. J. Phys. Chem. C.

[cit76] Ghosh S., Ourlin T., Mazarío J., Cayez S., Daccache S., Carrey J., Chaudret B. (2023). Fe@SiO2@Ni: An Iron-Based Composite Material for Magnetically Induced Hydrogenation Reactions in Gas and Solution Phases. Chem. Mater..

