# Supplementary material for: Induction heating applied to anisole HDO using formic acid as a hydrogen source
Source: Catal Sci Technol. 2025 Oct 8;16(1):302–15. doi: 10.1039/d5cy00707k (PMC12641493; doi:10.1039/d5cy00707k)
Supplement: CY-016-D5CY00707K-s001 [file CY-016-D5CY00707K-s001.pdf]

Supplementary Information (SI) for Catalysis Science & Technology.
This journal is © The Royal Society of Chemistry 2025

Supporting information

Induction heating applied to anisole HDO using Formic Acid as hydrogen source

Verónica Naharro-Ovejero[a], Mónica Dhanjani [b] Gorka Salas[b,c,d], Ana Belén Dongil [a*]

[a] Instituto de Catálisis y Petroleoquímica (CSIC), Campus Universitario de Cantoblanco, 28049 Madrid, Spain
a.dongil@csic.es

[b] Instituto Madrileño de Estudios Avanzados en Nanociencia, Campus Universitario de Cantoblanco, 28049 Madrid, Spain

[c] Unidad de Nanomateriales Avanzados, IMDEA Nanociencia (Unidad de I+D+I Asociada al Instituto de Ciencia de Materiales de Madrid, CSIC), 28019 Madrid, Spain

[d] Unidad Asociada de Nanobiotecnología (CNB-CSIC e IMDEA Nanociencia), 28049 Madrid, Spain

Table S1. Generated H_2 in the decomposition of formic acid (FA) reaction.

ANISOLE (mol)	$8.82x10^{-6}$
FA fed (mol)	$5.29x10^{-5}$

	Generated H_2 (mol/min)	Conversion (%)	CO_2 Selectivity (%)	H_2/ANIS.	CO (mol/min)
(C) Heating - ReO_x/G (HDO)	$4.23x10^{-5}$	100	80	4.8	$1.07x10^{-5}$
(C) Heating - ReO_x/G	$4.45x10^{-5}$	100	84	5.0	$8.47x10^{-6}$
(M) Heating - ReO_x/G + Fe_3O_4	$4.02x10^{-5}$	100	76	4.6	$1.27x10^{-5}$
(M) Heating - ReO_x/G + Fe_3O_4 (HDO)	$4.23x10^{-5}$	100	80	4.8	$1.06x10^{-5}$
(M) Heating - ReO_x/G + FeCo@C_{HT} (HDO)	$4.76x10^{-5}$	100	90	5.4	$5.29x10^{-6}$

Table S2. XPS atomic ratio (Re/C) and TXRF results of ReO_x/G spent catalyst after HDO reaction with conventional heating after using FA and H_2 at 320°C.

Sample	TXRF analysis	XPS ratio[1]
	Re (% wt.)	Re/C
ReO_x/G-FA(s)	10.4	$8.9x10^{-3}$
ReO_x/G-H_2(s)	-	$8.4x10^{-3}$

[1] Re/C of fresh ReOx/C catalyst $6.7x10^{-3}$

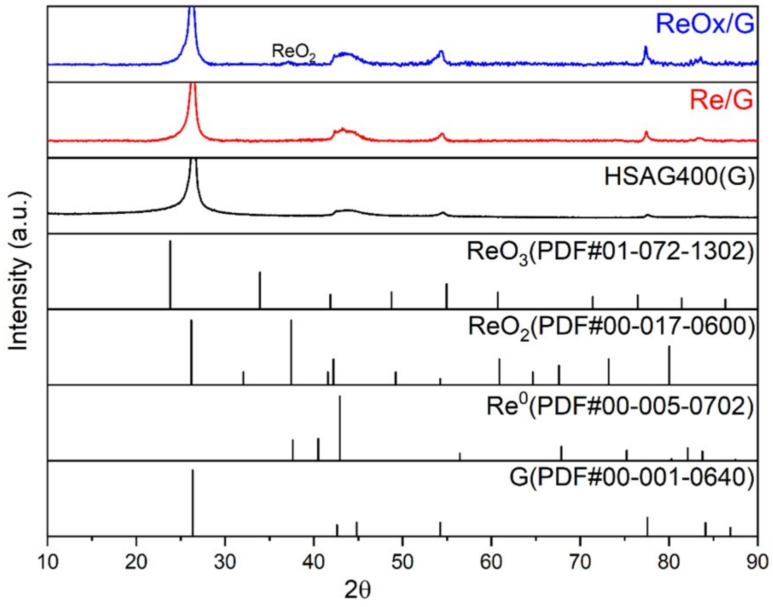

Fig. S1. XRD patterns of ReO_x/G and Re/G catalysts. ★ReO_2.

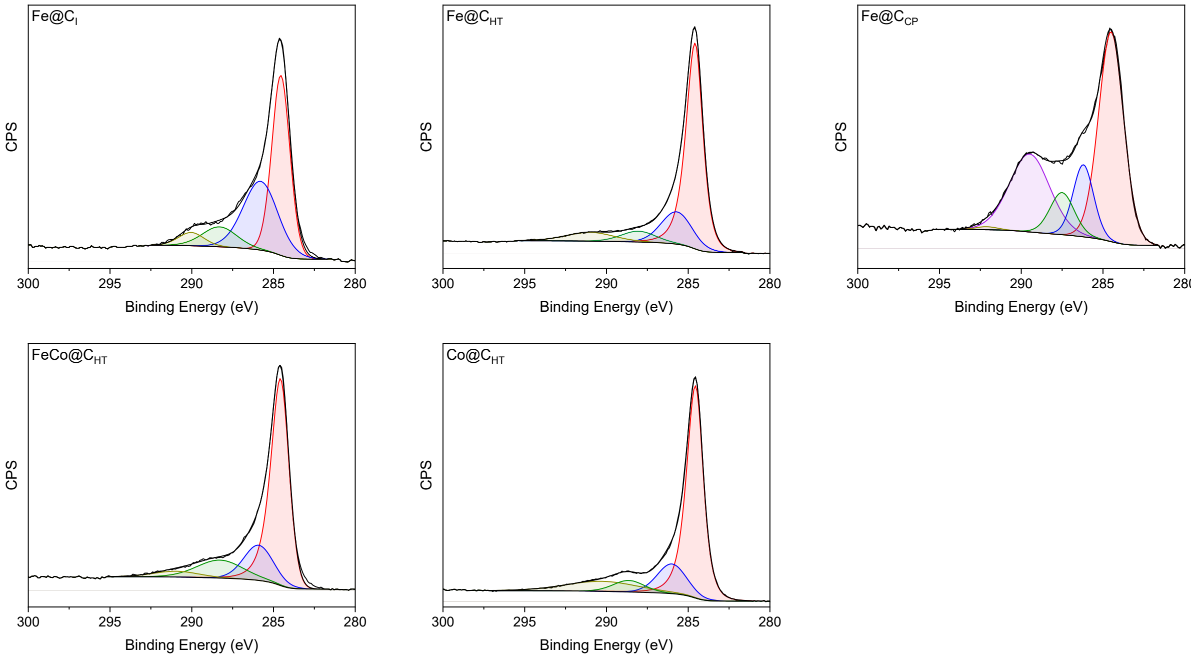

Fig. S2. XPS of the C 1s region for the Fe@C$_{HT}$, FeCo@C$_{HT}$, Fe@C$_{CP}$, Fe@C$_I$ and Co@C$_{HT}$ MNPs.

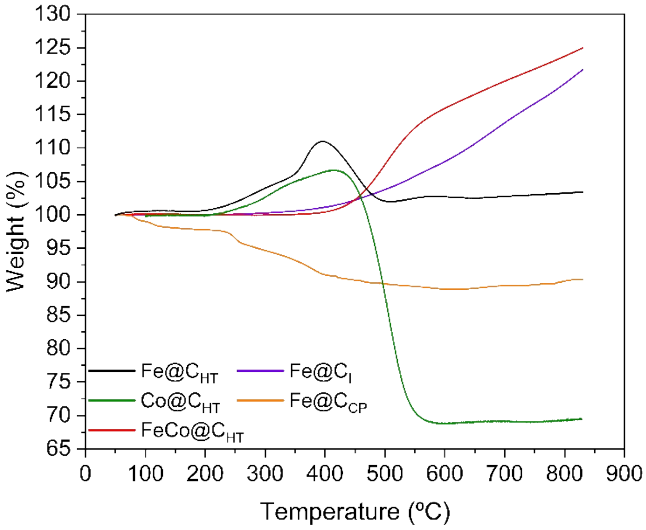

Fig. S3. TGA curves for Fe@C$_{HT}$, FeCo@C$_{HT}$, Fe@C$_{CP}$, Fe@C$_I$ and Co@C$_{HT}$ MNPs.

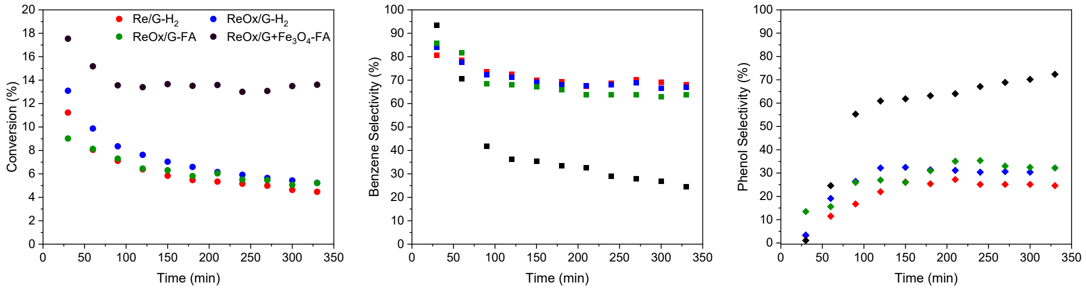

Figure S4. Conversion and selectivity to benzene (squares) and phenol (diamond) as a function of time during Anisole HDO over Re/G and ReO_x/G catalysts using H_2 or Formic Acid. Reaction conditions: Conventional heating 2.7% vol. Anisole, 320 °C. Colors: (●) ReO_x+Fe3O4-FA; (●) $ReO_x/G-H_2$; (●) ReO_x/G-FA; (●) $Re/G-H_2$.

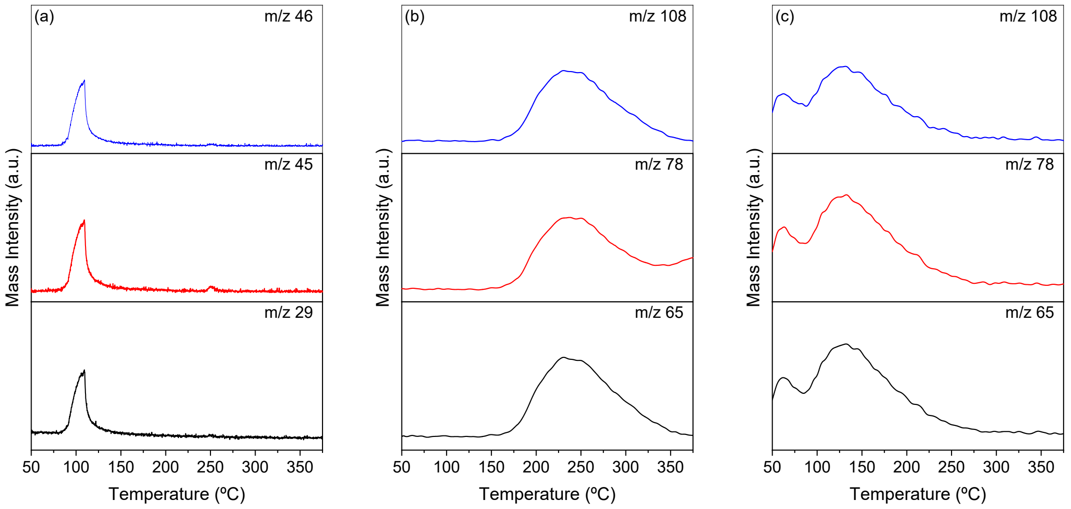

Fig. S5. Spent-TPD of (a), (b) ReO_x/G in the HDO of Anisole Reaction with FA and (c) ReO_x/G in the HDO of Anisole Reaction with H_2. Mass: formic acid (m/z: 29, 45, 46) and anisole (m/z: 65, 78, 108).

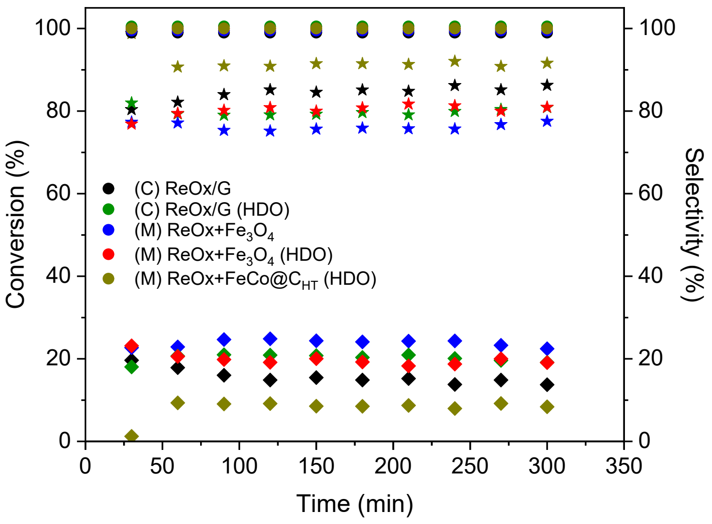

Fig. S6. Magnetic (M) and conventional (C) heating experiments for formic acid decomposition using, ReO_x/G and selected MNPs without (FA decomposition only) or with the presence of anisole (during anisole HDO). FA conversion

(circles), selectivity to CO_2 (stars) and selectivity to CO (diamond) vs time. Reaction conditions: 2.7% vol FA, 285 mg; 320 °C, 100 kHz, 26 mT (Fe_3O_4), 16 mT (FeCo@C_{HT}).

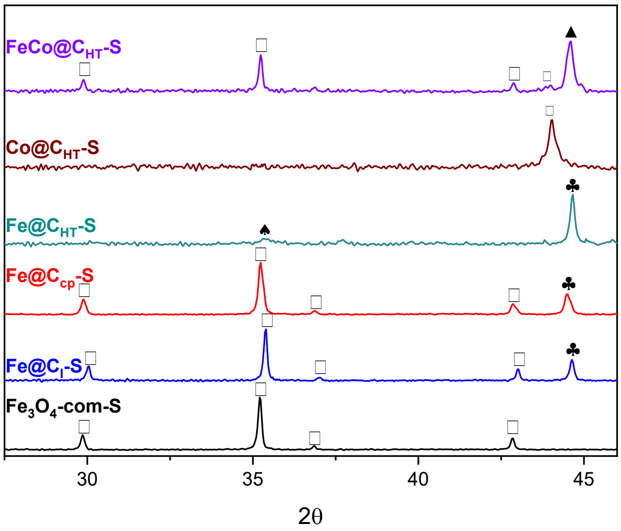

Fig. S7. XRD patterns of spent MNPs. ★Fe_3O_4 ♠FeO ♣Fe(0) ✖ Co(0) ▲ FeCo.
